# Supplementary material for: Small Molecules Promote the Rapid Generation of Dental Epithelial Cells from Human-Induced Pluripotent Stem Cells
Source: Int J Mol Sci. 2024 Apr 9;25(8):4138. doi: 10.3390/ijms25084138 (PMC11049943; doi:10.3390/ijms25084138)
Supplement: Supplementary file 1 [file ijms-25-04138-s001.zip › ijms-2907312-supplementary.pdf]

Table S1: Primers

Gene	Sequence	Rationale
GAPDH		
Forward	CTCCTCCACCTT TGACGCTG	Housekeeping
Reverse	TCCTCTTGTGCTCTTGCTG	
TFAP2A		
Forward	TAAAGCTGCCAACGTTACCC	Non-neural ectoderm
Reverse	GCACACGTACCCAAAGTCC	
DLX3		
Forward	AGCCTCCTACCGGCAATAC	Non-neural ectoderm
Reverse	TTCCGGCTCCTCCTTCAC	
DLX5		
Forward	GCTAGCTCCTACCACCAGTAC	Non-neural ectoderm
Reverse	GGTTTGCCATTCACCATTCTCA	
OCT4		
Forward	ACCCCTGGTGCCGTGAA	Pluripotency
Reverse	GGCTGAATACCT TCCCAAATA	
K18		
Forward	CTCTGTCCAGGCGCCCAGCT	Surface ectoderm
Reverse	CCCCTGAAGCTGGTGGAG	
SOX1		
Forward	CAACTCGCAGGGCTACATGA	Neural ectoderm
Reverse	CTCCGACTTCACCAGAGAGC	
SIX1		
Forward	GGTTTAAGAACCGGAGGCAAA	Pan-placodal ectoderm
Reverse	TGCTTGTTGGAGGAGGAGTTA	
Brachyury		
Forward	ATGAGCCTCGAATCCACATAGT	Mesoderm
Reverse	TCCTCGTTCTGATAAGCAGTCA	
FOXA2		
Forward	GAGCAGCAGCGGGCGAGTTA	Endoderm
Reverse	CCCAGGCCGGCGTTCATGTT	
EYA1		
Forward	ACAGCCGACGGGTCTTTAA	Pan-placodal ectoderm
Reverse	TTGGTCGTGGGCTGAAACTA	
PITX2		
Forward	AGTCCGGGTTTGGTTCAAGA	Dental epithelial and Oral epithelial
Reverse	TGGATAGGGAGGCGGATGTA	
PITX1		
Forward	GTGGCGTAAGCGCGAGCGTAA	Oral epithelial
Reverse	GACAGCGGGCTCATGGAGTTGAAG	
AMBN		
Forward	AGAGCACAGTGCATGTCAGC	Dental epithelial and Ameloblast
Reverse	AAGAACGGCACTGCAAAACT	
SP6		
Forward	GGACTTCTCGCAGGGCTATG	Dental epithelial and Ameloblast
Reverse	GCTGCAGGAGCTTGGAAAAG	

Table S2: Antibodies

Antibodies	Supplier	Indentifier
TFAP2A	Santa Cruz	sc-12726
OCT4	Santa Cruz	sc-5279
SIX1	Proteintech	10709-1-AP
PITX2	Santa Cruz	sc-390457
PITX1	Santa Cruz	sc-271435
AMBN	Santa Cruz	sc-271012
AMELX	Santa Cruz	sc-365284
SOX2	Abclonal	A0561
Ki67	Abmart	TW0001

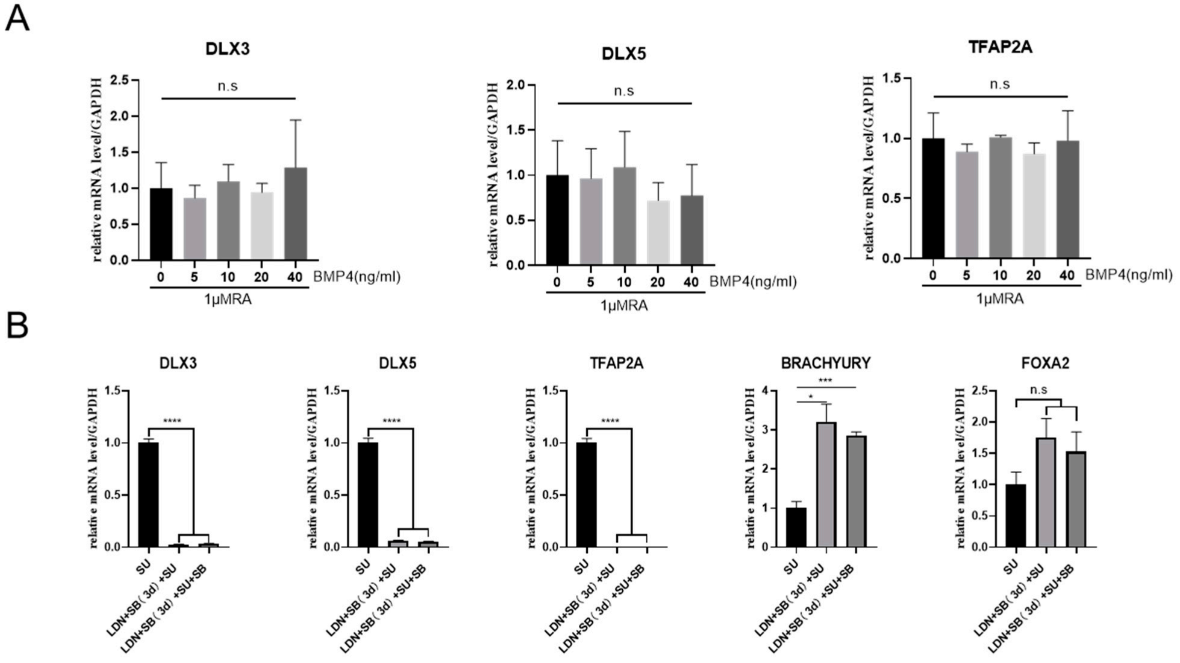

Figure S1. Selection of induction conditions for acquisition of NNE fate. **A**. qRT-PCR analysis revealed the expression of non-neural ectoderm (NNE) markers TFAP2A, DLX3, and DLX5 in response to the stimulation of 1 μM RA in combination with BMP4 at various concentrations. **B**. qRT-PCR analysis revealed the expression of TFAP2A, DLX3, DLX5, Brachyury, and FOXA2 subsequent to the addition of LDN and SB during the early stages of differentiation. Data shown are mean ± SEM of three independent experiments (three technical replicates per biological replicate). *, $p < 0.05$, ***, $p < 0.001$, ****, $p < 0.0001$ and n.s, not significant.
